# Life course socioeconomic position, intergenerational social mobility, and mortality among Brazilian public servants in the ELSA-Brasil cohort

**DOI:** 10.1590/0102-311XEN009625

**Published:** 2025-10-24

**Authors:** Jose Aparecido Soares Lopes, Luana Giatti, Lidyane do Valle Camelo, Luisa Campos Caldeira Brant, Rosane Harter Griep, Antonio Luis Ribeiro, Sandhi Maria Barreto

**Affiliations:** 1 Departamento de Assuntos Estudantis e Comunitários, Instituto Federal do Norte de Minas Gerais, Januária, Brasil.; 2 Faculdade de Medicina, Universidade Federal de Minas Gerais, Belo Horizonte, Brasil.; 3 Hospital das Clínicas/Empresa Brasileira de Serviços Hospitalares, Universidade Federal de Minas Gerais, Belo Horizonte, Brasil.; 4 Instituto Oswaldo Cruz, Fundação Oswaldo Cruz, Rio de Janeiro, Brasil.

**Keywords:** Socioeconomic Status, Mortality, Social Mobility, Cohort Studies, Posição Socioeconômica, Mortalidade, Mobilidade Social, Estudos de Coortes, Nível Socioeconómico, Mortalidad, Movilidad Social, Estudios de Cohortes

## Abstract

This study examined whether a low socioeconomic position over the course of one’s life, the accumulation of low socioeconomic position, and intergenerational social mobility are associated with all-cause mortality over a 15-year follow-up period, and whether these associations varied according to race/skin color. A prospective study was conducted with 13,652 participants from the *Brazilian Longitudinal Study of Adult Health* (ELSA-Brasil) cohort. The outcome was time to death from any cause. The explanatory variables were socioeconomic position in childhood (mother’s schooling), adolescence (head of household’s socio-occupational class), adulthood (participant’s schooling and socio-occupational class), cumulative socioeconomic position, and intergenerational social mobility. Cox proportional hazards models were adjusted for sociodemographic characteristics. The mortality rate was 4.9/1,000 person-years and was higher among men, older adults, Blacks, and those with a low socioeconomic position. After adjustments, low socioeconomic position in childhood, adolescence, and adulthood remained associated with higher mortality. The greatest accumulation of low socioeconomic position across life (HR = 2.02; 95%CI: 1.64-2.48, 4th vs. 1st quartile), as well as downward and stable low educational and socio-occupational trajectories, were also associated with higher mortality. To a lesser degree, an upward socio-occupational trajectory (vs. stable high) increased the risk of death. No multiplicative interaction was found between socioeconomic position and race/skin color regarding risk of death. Lifelong exposure to socioeconomic disadvantages throughout the course of life as well as the accumulation of adverse social experiences and unfavorable intergenerational educational and socio-occupational mobility, increased the risk of mortality, demonstrating the long-term effect of a low socioeconomic position, especially with prolonged exposure.

## Abstract (pt)

O presente estudo examinou se uma baixa posição socioeconômica ao longo da vida, o acúmulo de baixa PSE e a mobilidade social intergeracional se associaram à mortalidade por todas as causas em um período de acompanhamento de 15 anos e se essas associações variaram de acordo com raça/cor. Um estudo prospectivo foi conduzido com 13.652 participantes do *Estudo Longitudinal de Saúde do Adulto* (ELSA-Brasil). O desfecho foi o tempo até a morte por qualquer causa. As variáveis explicativas foram posição socioeconômica na infância (escolaridade da mãe), na adolescência (classe socioocupacional do chefe da família) e na vida adulta (escolaridade e classe socioocupacional do participante), bem como a posição socioeconômica cumulativa e a mobilidade social intergeracional. Os modelos de riscos proporcionais de Cox foram ajustados por características sociodemográficas. A mortalidade foi de 4,9/1.000 pessoas-ano e foi maior nos homens, idosos, negros e aqueles com baixa posição socioeconômica. Após os ajustes, baixa posição socioeconômica na infância, na adolescência e na vida adulta permaneceu associado à maior mortalidade. O maior acúmulo de baixa posição socioeconômica ao longo da vida (HR = 2,02; IC95%: 1,64-2,48, 4º *vs.* 1º quartil), bem como trajetórias educacionais e socioocupacionais descendentes e estáveis-baixas, também se associaram a maior mortalidade. Em menor grau, uma trajetória sócio-ocupacional ascendente (*vs.* alta estável) aumentou o risco de morte. Não foi encontrada interação multiplicativa entre posição socioeconômica e raça/cor no risco de morte. A exposição a desvantagens socioeconômicas ao longo da vida, o acúmulo de experiências sociais negativas e a mobilidade sócio-ocupacional intergeracional desfavorável aumentaram o risco de morte, demonstrando o efeito a longo prazo de uma posição socioeconômica baixa, especialmente com exposição prolongada.

## Abstract (es)

Este estudio analizó si el bajo nivel socioeconómico del curso de vida, la baja acumulación de nivel socioeconómico y la movilidad social intergeneracional están asociados con la mortalidad general durante 15 años de seguimiento, y si estas asociaciones variaron según la raza/color. Se realizó un estudio prospectivo con 13.652 participantes de la cohorte *Estudio Longitudinal de Salud del Adulto en Brasil* (ELSA-Brasil). El resultado fue el tiempo de muerte por cualquier causa. Las variables explicativas fueron nivel socioeconómico en la infancia (nivel educativo materno), juventud (clase sociolaboral del jefe de hogar), vida adulta (nivel educativo y clase sociolaboral del participante), PSE acumulativa y movilidad social intergeneracional. Se utilizaron modelos de riesgos proporcionales de Cox ajustados por características sociodemográficas. La mortalidad fue de 4,9/1.000 personas-año, más alta en hombres, ancianos, negros y aquellos con baja nivel socioeconómico. Después de los ajustes, una baja nivel socioeconómico durante la infancia, la juventud y la edad adulta permaneció asociada con una mayor mortalidad. La mayor acumulación de baja nivel socioeconómico en la vida (HR = 2,02; IC95%: 1,64-2,48, 4º cuartil *vs.* 1º) y las trayectorias educativas y sociolaborales descendentes y baja estable también se asociaron con una mayor mortalidad. En menor medida, la trayectoria socioocupacional ascendente (frente a la alta estable) aumentó el riesgo de muerte. No hubo interacción multiplicativa entre nivel socioeconómico y raza/color en el riesgo de muerte. La exposición a desventajas socioeconómicas en el curso de la vida, así como la acumulación de experiencias sociales negativas y la movilidad intergeneracional educativa y socioprofesional desfavorable aumentaron el riesgo de mortalidad, lo que demuestra el efecto a largo plazo de baja nivel socioeconómico en la salud, especialmente la exposición más prolongada.

## Introduction

Socioeconomic inequalities in mortality have been increasing in recent years and constitute a global public health concern ^1^. Low socioeconomic status affects health in ways comparable to major risk factors for mortality, contributing to higher morbidity and premature mortality among the poorest and most socially vulnerable groups ^1^ ^,^ ^2^. Despite this, socioeconomic circumstances remain neglected by mortality reduction policies ^2^.

Socioeconomic position, usually studied by analyzing income and education, indicates an individual’s or group’s status within the social structure and is especially useful to reveal health or social inequities ^3^ ^,^ ^4^. There is a consistent inverse socioeconomic gradient between socioeconomic position indicators in childhood and adulthood and higher all-cause mortality ^1^, cause-specific mortality ^5^ ^,^ ^6^, and premature death ^2^. Furthermore, evidence suggests that social mobility is associated with the risk of death, with individuals who worsen or remain in a low socioeconomic position throughout life facing worse outcomes ^7^ ^,^ ^8^. However, it is unclear whether mobility itself, regardless of its direction, has a direct impact on health risks ^9^. Moreover, studies show that black individuals are more frequently exposed to socioeconomic disadvantages, have less upward social mobility ^10^ ^,^ ^11^, and die earlier when compared to white individuals ^12^.

The approach to socioeconomic position in different life stages is grounded in life course epidemiology. This framework, by using theoretical models − such as critical and sensitive periods, accumulation of risks, and social mobility − seeks to understand how socioeconomic adversities during different development stages and across generations affects illness and death during adulthood ^13^ ^,^ ^14^. These models recognize socioeconomic factors as fundamental causes of illness and death, as they shape access to resources for health protection, including knowledge, financial resources, power, prestige, and social networks ^15^.

Social mobility refers to changes in individuals’ or families’ positions within a society’s stratification system over time, whether across generations or throughout the life course. It is typically assessed using indicators such as social class, income, and education, and involves tracking trajectories of stability, upward, or downward movement. Low social mobility and the accumulation of socioeconomic adversities across the life course are characteristic of unequal societies with few opportunities for social mobility. In Brazil, an individual born among the bottom 10% of the income distribution may take up to nine generations to reach the national average income. Among the 30 countries from the Organisation for Economic Co-operation and Development (OECD), Brazil outperforms only Colombia ^16^. This scenario is even worse for women and black people when compared to men and white people in Brazil ^17^.

Studies on life course socioeconomic position and mortality remain scarce in low- and middle-income countries, and no longitudinal study with this approach was found in Brazil. However, findings from the *Brazilian Longitudinal Study of Adult Health* (ELSA-Brasil) cohort have shown that intragenerational downward social mobility is associated with increased blood pressure ^18^, and that persistent low socioeconomic position across generations and accumulation of socioeconomic disadvantages throughout life are linked to a higher risk of arterial hypertension ^19^.

This study investigated whether low socioeconomic position during one’s life course − especially the accumulation of exposure to low socioeconomic position and unfavorable intergenerational social mobility − is, in fact, associated with higher all-cause mortality over approximately 15 years of follow-up in a multicenter and multiethnic Brazilian cohort. Additionally, our study verified whether these associations vary by race/skin color. We hypothesized that: (1) exposure to low socioeconomic position at all stages of one’s life is associated with higher all-cause mortality; (2) greater accumulation of low socioeconomic position exposure throughout life predicts higher all-cause mortality; (3) unfavorable intergenerational social mobility predicts higher all-cause mortality; and (4) the strength of these associations with life course socioeconomic position and intergenerational social mobility is greater among black individuals.

## Methods

### Study type and population

**Figure 1:**
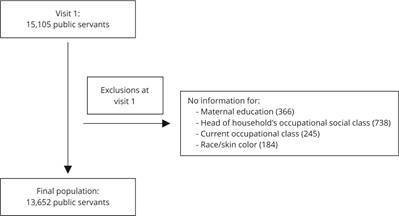
Study population flowchart.

This is a longitudinal study using data from ELSA-Brasil, a multicenter cohort study conducted with 15,105 active and retired civil servants aged 35-74 years at visit 1 (2008-2010), working in educational and research institutions located in six Brazilian capitals. Participants completed structured questionnaires via face-to-face interviews and underwent clinical and laboratory tests ^20^. Data was collected by trained and certified professionals, following a strict quality assurance and control protocol. The study design and cohort profile are described in previous publications ^21^ ^,^ ^22^. The research protocol was approved by the Research Ethics Committees of all participating institutions (Federal University of Minas Gerais − UFMG, University of São Paulo − USP, Federal University of Rio Grande do Sul − UFRGS, Federal University of Espírito Santo − UFES, Federal University of Bahia − UFBA, and Oswaldo Cruz Foundation − FIOCRUZ), and all participants signed the informed consent form. For this study, all cohort participants (N = 15,105) were initially eligible. Individuals with missing data for maternal education (n = 157), head of household’s occupational social class (n = 738), participant’s current occupational class (n = 245), and race/skin color (n = 184) were excluded. Considering overlapping characteristics, the final study population totaled 13,652 individuals, representing 90.4% of those eligible (Figure 1).

### Study variables

#### Response variable

The response variable in this study was time to death. Total person-time of follow-up corresponds to the sum of each individual person-time obtained by the difference in years from the cohort entry date and the earliest of the following events: date of death, study withdrawal, or the end of follow-up on April 21, 2024. Data on mortality were obtained via annual follow-up calls and search on hospital records ^23^. The underlying cause of death was defined according to the 10th revision of the International Classification of Diseases (ICD-10) and obtained from death certificates, hospital admissions records, or cross-referencing with data from the Brazilian Mortality Information System (SIM) of the Brazilian Ministry of Health ^24^.

#### Explanatory variables

a) Childhood socioeconomic position: assessed by maternal educational level, based on the question “What is your mother’s educational level?”. Responses were originally categorized as postgraduate, complete higher education, incomplete higher education, complete high school, incomplete high school, complete elementary school, incomplete elementary school, and never studied. In our analysis, these categories were grouped as “never studied”, “incomplete elementary school”, “complete elementary school”, and “high school or higher”.

b) Youth socioeconomic position: defined by the head of household’s occupational class at the time the participant started working, which was 17 years old on average. Occupational social class was obtained by detailed analysis of the described work activities, considering the relationship between the typical income for a given occupation in the labor market and the expected income according to educational requirements for that occupation. Occupational social class was originally categorized as high-upper, high-low, middle-upper, middle-middle, middle-low, low-high and low-low ^25^. In our study, they were grouped as “high” (high-upper, high-low), “middle-upper”, “middle” (middle-middle and middle-low), and “low” (low-high and low-low).

c) Adulthood socioeconomic position: participant’s educational level (with the same categories of maternal educational level): categorized as “incomplete elementary school,” “complete elementary school,” “high school,” and “higher education or more” − and the participant’s current occupational social class, categorized similarly to that of the head of household. Both educational level and occupational social class were relative to the time of entry into the cohort.

d) Cumulative socioeconomic position: indicates the accumulation of exposure to low socioeconomic position during one’s life, considering maternal education, head of household’s, and individuals’ social class. First, we attributed a note to each category level as shown: maternal educational level (≥ 15 years of study = 0; 11-14 years of study = 1; 8-10 years of study = 2; 1-7 years of study = 3; never studied = 4); head of household’s occupational class (high = 0; middle-upper = 1; middle-middle = 2; middle-low = 3; low = 4); and individual’s occupational social class (high = 0; middle-upper = 1; middle-middle = 2; middle-low = 3; low = 4). Then we added them up to obtain the total score of the cumulative socioeconomic position, which ranged from 0 to 12 points. Finaly, we grouped the score into quartiles, with the 1st quartile representing better socioeconomic position and the 4th the worse socioeconomic position.

e) Intergenerational educational social mobility: assessed using different cutoffs for maternal and participants’ educational level, due to their distinct distribution. Maternal education was defined as “high” (complete elementary school or higher) and “low” (incomplete elementary school or less), and the participant’s educational level was categorized as “high” (higher education or more) and “low” (high school or less). These two variables were then compared to define four mobility categories: high-stable (high education for both mother and participant), upward (low maternal education and high participant education), downward (high maternal education and low participant education), and low-stable (low education for both mother and participant).

f) Intergenerational occupational social mobility: an occupational mobility matrix was created crossing the occupational social class of the head of household and of the participant. Both variables had seven categories (low-low, low-high, middle-low, middle-middle, middle-upper, high-low, and high-upper), totaling 64 possible occupational trajectories ^18^. These trajectories were then grouped into four categories − high-stable, upward, downward, and low-stable − according to the cutoffs defined in Supplementary Material (Figure S1; https://cadernos.ensp.fiocruz.br/static//arquivo/suppl-e00009625_7579.pdf).

### Covariables

**Figure 2:**
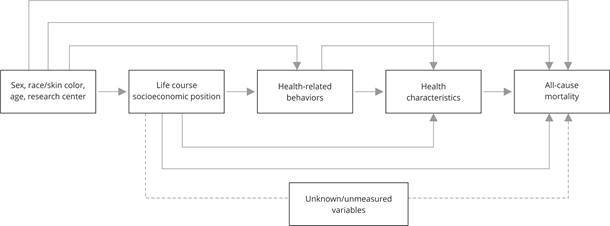
Theoretical-operational model.

Sociodemographic characteristics − sex, age (continuous, in years), and self-reported race/skin color (categorized as white, black, mixed-race, Asian, and Indigenous) − precede socioeconomic position at all life stages and were included in the analyses as potential confounders. The research center (São Paulo, Minas Gerais, Rio Grande do Sul, Rio de Janeiro, Bahia, and Espírito Santo) was also included in the adjustments as it reflects regional sociocultural differences that may affect the associations studied. Health behaviors and characteristics were not included in the analyses, as they are considered mediators in the association between life-course socioeconomic position and all-cause mortality (Figure 2). All covariates were obtained at visit 1.

### Data analysis

The incidence density of deaths was obtained by dividing the number of deaths by the total person-years at risk, and was described according to the characteristics of the study population. Unadjusted survival curves according to socioeconomic position indicators were estimated using the Kaplan-Meier method, and differences between curves were assessed using the log-rank test.

The Cox proportional hazards model was used to investigate the magnitude of the association between each life course socioeconomic position separately and the risk of death from all causes. Crude hazard ratios (HR) and 95% confidence intervals (95%CI) were estimated (Model 0); subsequently, age, sex, and research center were included (Model 1). Finally, race/skin color was included in the fully adjusted model (Model 2). The proportional hazards assumption was assessed using Schoenfeld residuals, and the assumption was met in all models. All covariates were maintained in the final model regardless of statistical significance.

Multiplicative interaction terms between socioeconomic position indicators and race/skin color were added to the models to verify race/skin color differentials in the associations found. Additionally, we tested for linear trend by including the ordinal exposure variables as continuous terms in the Cox proportional hazards models. This approach enabled a statistical evaluation of linear trends across ordered categories while adjusting for potential confounders. A 5% significance level was adopted for all analyses, which were conducted using Stata 14.0 (https://www.stata.com).

## Results

Of the 13,652 participants, most were female (54.7%) and self-identified as white (52.3%). The median (IQR) age was 51 years (IQR = 45-58) (Table 1).

The median follow-up time was 13.8 years (IQR = 13.0-14.1). A total of 886 deaths from all causes were recorded, corresponding to an incidence density of 4.9 deaths per 1,000 person-years. Higher risk of death was observed among men, older individuals, self-declared blacks, and those with a lower socioeconomic position (Table 1).

**Table 1:** Distribution of the study population and mortality according to sociodemographic characteristics, research center, socioeconomic position, and intergenerational social mobility indicators during the 15 years of follow-up. *Brazilian Longitudinal Study of Adult Health* (ELSA-Brasil, N = 13,652), 2008-2024.

Characteristics *	n (%)	Number of deaths due to all causes	Person-years at risk	Incidence density/1,000 person-years (95%CI)
Sex				
Male	6,180 (45.3)	525	80,346.2	6.5 (6.6-7.1)
Female	7,472 (54.7)	361	100,151.0	3.6 (3.3-4.0)
Age (years) [median (IQR)]	51 (45-58)	-	-	-
Race/Skin color				
White	7,146 (52.3)	386	94,757.6	4.1 (3.7-4.5)
Mixed-race	3,848 (28.2)	276	50,783.7	5.4 (4.8-6.1)
Black	2,170 (15.9)	188	28,506.5	6.6 (5.7-7.6)
Asian	342 (2.5)	22	4,553.3	4.8 (3.2-7.3)
Indigenous	146 (1.0)	14	1,896.0	7.4 (4.4-12.5)
Research center				
São Paulo	4,600 (33.7)	299	61,631.4	4.9 (4.3-5.4)
Espírito Santo	958 (7.0)	65	12,836.5	5.1 (4.0-6.5)
Minas Gerais	2,934 (21.5)	170	38,713.7	4.4 (3.8-5.1)
Rio de Janeiro	1,593 (11.7)	89	20,891.5	4.3 (3.5-5.2)
Rio Grande do Sul	1,772 (13.0)	114	22,621.9	5.0 (4.2-6.1)
Bahia	1,795 (13.2)	149	23,802.1	6.3 (5.3-7.4)
Childhood socioeconomic position				
Maternal education				
High school or higher	3,225 (23.6)	144	42,738.9	3.4 (2.9-4.0)
Complete elementary school	2,640 (19.3)	150	35,102.2	4.3 (3.6-5.0)
Incomplete elementary school	5,848 (42.8)	394	77,702.6	5.1 (4.6-5.6)
Never studied	1,939 (14.2)	198	24,953.4	7.9 (6.9-9.1)
Youth socioeconomic position				
Head of household’s occupational social class				
High	2,984 (21.9)	158	39,531.4	4.0 (3.4-4.7)
Middle-upper	1,329 (9.7)	53	17,707.3	3.0 (2.3-3.9)
Middle	2,546 (18.7)	171	33,579.7	5.1 (4.4-5.9)
Low	6,793 (49.8)	504	89,678.7	5.6 (5.2-6.1)
Adulthood socioeconomic position				
Participant’s education				
Higher education	7,263 (53.2)	325	96,920.4	3.4 (3.0-3.7)
High school	4,739 (34.7)	322	62,978.6	5.1 (4.6-5.7)
Complete elementary school	901 (6.6)	116	11,498.2	10.1 (8.4-12.1)
Incomplete elementary school	749 (5.5)	123	9,099.8	13.5 (11.3-16.1)
Current occupational social class				
High	4,627 (33.9)	235	61,332.9	3.8 (3.4-4.4)
Middle-upper	3,267 (23.9)	154	43,812.5	3.5 (3.0-4.1)
Middle	2,479 (18.2)	174	32,935.3	5.3 (4.6-6.1)
Low	3,279 (24.0)	323	42,416.4	7.6 (6.8-8.5)
Accumulation of low socioeconomic position (quartile)				
1st	4,466 (32.7)	194	59,278.4	3.3 (2.8-3.8)
2nd	3,210 (23.5)	195	42,769.2	4.6 (4.0-5.2)
3rd	3,544 (26.0)	238	47,130.0	5.0 (4.4-5.7)
4th	2,432 (17.8)	259	31,319.5	8.3 (7.3-9.3)
Intergenerational mobility in the occupational social class				
High-stable	3,175 (23.4)	137	42,129.8	3.3 (2.8-3.8)
Upward	4,157 (30.5)	225	55,501.2	4.1 (3.6-4.6)
Downward	1,761 (12.9)	115	23,281.9	4.9 (4.1-5.9)
Low-stable	4,559 (33.4)	409	59,584.2	6.9 (6.2-7.6)
Intergenerational mobility in educational level				
High-stable	4,248 (31.1)	179	56,492.6	3.2 (2.7-3.7)
Upward	3,015 (22.1)	146	40,427.8	3.6 (3.1-4.2)
Downward	1,617 (11.8)	115	21,348.4	5.4 (4.5-6.5)
Low-stable	4,772 (35.0)	446	62,228.2	7.2 (6.5-7.9)

95%CI: 95% confidence interval.

Note: some frequencies may add up to 100.1% or 99.9% due to rounding.

* Population characteristics at visit 1.

**Figure 3:**
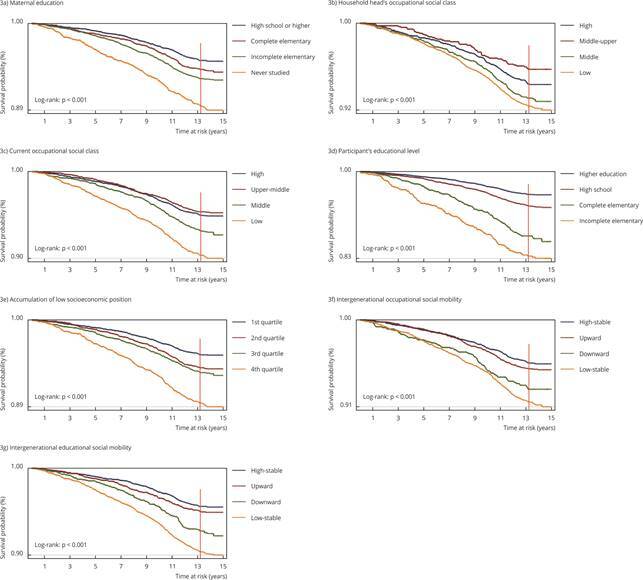
Kaplan-Meier survival curves for socioeconomic position indicators and all-cause mortality.

Figures 3a, 3b, 3c, 3d, 3e, 3f and 3g show the unadjusted Kaplan-Meier survival curves for socioeconomic position indicators. Survival probability was lower for almost all socioeconomic position categories below the reference group (log-rank test: p < 0.001 for all analyses) (Figure 3).

After adjustment for race/skin color, low childhood socioeconomic position was associated with higher risk of death from all causes. Participants whose mothers had never attended school (vs. high school or higher education) presented a 41% higher risk of death (95%CI: 1.12-1.77) (Model 2, Table 2). Among the youth, having a head of household with middle or low occupational social class (vs. high) was also associated with higher mortality, even after adjustment for race/skin color (HR = 1.42; 95%CI: 1.14-1.77 and HR = 1.39; 95%CI: 1.15-1.67, respectively) (Model 2, Table 2). The risk of death was also higher among participants with lower educational levels, showing a clear dose-response gradient. After considering race/skin color, participants with incomplete elementary school had more than double the risk of death compared to those with higher education or more (HR = 2.37; 95%CI: 1.89-2.97) (Model 2, Table 2). Current occupational social class was also associated with a higher risk of death across all categories below the upper class, following a dose-response gradient. Even after adjusting for race/race, individuals in the lower class had almost twice the risk of dying from all causes (HR = 1.99; 95%CI: 1.66-2.39) (Model 2, Table 2).

**Table 2:** Hazard ratio (HR) and 95% confidence interval (95%CI) for all-cause mortality during approximately 15 years of follow-up according to indicators of life course socioeconomic position. *Brazilian Longitudinal Study of Adult Health* (ELSA-Brasil, N = 13,652), 2008-2024.

Indicator	Model 0	Model 1	Model 2
HR (95%CI)	HR (95%CI)	HR (95%CI)
Socioeconomic position during childhood			
Maternal education			
High school or higher	1.00	1.00	1.00
Complete elementary school	1.27 (1.01-1.60)	1.20 (1.00-1.51)	1.10 (0.90-1.39)
Incomplete elementary school	1.51 (1.25-1.83)	1.48 (1.23-1.80)	1.29 (1.06-1.57)
Never studied	2.38 (1.92-2.95)	1.81 (1.45-2.24)	1.41 (1.12-1.77)
p-value for linear trend	< 0.001	< 0.001	< 0.001
Socioeconomic position during youth			
Head of household’s occupational social class			
High	1.00	1.00	1.00
Middle-upper	0.75 (0.55-1.02)	0.86 (0.63-1.18)	0.84 (0.62-1.15)
Middle	1.28 (1.03-1.59)	1.59 (1.28-1.97)	1.42 (1.14-1.77)
Low	1.41 (1.18-1.69)	1.63 (1.36-1.95)	1.39 (1.15-1.67)
p-value for linear trend	< 0.001	< 0.001	< 0.001
Socioeconomic position during adulthood			
Participant’s education			
Higher education	1.00	1.00	1.00
High school	1.54 (1.32-1.79)	1.91 (1.63-2.23)	1.74 (1.47-2.05)
Complete elementary school	3.04 (2.46-3.75)	2.42 (1.95-3.00)	2.17 (1.73-2.71)
Incomplete elementary school	4.09 (3.32-5.03)	2.71 (2.19-3.35)	2.37 (1.89-2.97)
p-value for linear trend	< 0.001	< 0.001	< 0.001
Current occupational social class			
High	1.00	1.00	1.00
Middle-upper	0.92 (0.75-1.13)	1.59 (1.29-1.97)	1.48 (1.19-1.83)
Middle	1.39 (1.14-1.69)	2.05 (1.67-2.51)	1.81 (1.46-2.23)
Low	2.00 (1.70-2.37)	2.30 (1.94-2.72)	1.99 (1.66-2.39)
p-value for linear trend	< 0.001	< 0.001	< 0.001
Accumulation of low socioeconomic position (quartile)			
1st	1.00	1.00	1.00
2nd	1.40 (1.15-1.71)	1.56 (1.28-1.91)	1.47 (1.20-1.80)
3rd	1.56 (1.29-1.88)	2.00 (1.65-2.43)	1.76 (1.44-2.15)
4th	2.55 (2.12-3.08)	2.39 (1,98-2.89)	2.02 (1.64-2.48)
p-value for linear trend	< 0.001	< 0.001	< 0.001
Intergenerational mobility in the occupational social class			
High-stable	1.00	1.00	1.00
Upward	1.25 (1.01-1.55)	1.48 (1.19-1.83)	1.38 (1.11-1.71)
Downward	1.53 (1.19-1.96)	1.94 (1.51-2.49)	1.78 (1.38-2.30)
Low-stable	2.13 (1.76-2.59)	2.46 (2.02-2.99)	2.08 (1.68-2.56)
p-value for linear trend	< 0.001	< 0.001	< 0.001
Intergenerational mobility in the educational level			
High-stable	1.00	1.00	1.00
Upward	1.14 (0.92-1.42)	1.13 (0.91-1.41)	1.09 (0.87-1.35)
Downward	1.71 (1.35-2.16)	2.16 (1.71-2.74)	1.97 (1.55-2.51)
Low-stable	2.28 (1.92-2.72)	2.29 (1.92-2.73)	2.00 (1.65-2.42)
p-value for linear trend	< 0.001	< 0.001	< 0.001

Note: Model 0: univariate analysis; Model 1: age, sex, and research center; Model 2: age, sex, research center, and race/skin color.

The accumulation of socioeconomic disadvantages during one’s lifetime was shown to be a strong predictor of all-cause mortality. Compared to those least exposed (1st quartile), individuals in the 2nd, 3rd, and 4th quartiles had a 47% (HR = 1.47; 95%CI: 1.20-1.80), 76% (HR = 1.76; 95%CI: 1.44-2.15), and 102% (HR = 2.02; 95%CI: 1.64-2.48) higher risk of death, demonstrating a clear dose-response gradient (Model 2, Table 2). 

Regarding intergenerational social mobility in occupational class, ascending, descending, or remaining in low socioeconomic position between generations (vs. remaining in the upper class) was associated with an increased risk of death (HR = 1.38; 95%CI: 1.11-1.71; HR = 1.78; 95%CI: 1.38-2.30; HR = 2.08; 95%CI: 1.68-2.56, respectively), considering participants’ race/skin color. As for intergenerational educational mobility, the downward and low-stable categories were also associated with a higher risk of death from all causes, even after full adjustment (HR = 1.97; 95%CI: 1.55-2.51 and HR = 2.00; 95%CI: 1.65-2.42) (Model 2, Table 2). 

We found no evidence of statistical interaction between socioeconomic position or intergenerational social mobility indicators studied and race/skin color concerning risk of death (p > 0.05). We also verified statistical interaction using a dichotomized race/skin color variable (white versus black and mixed-race), but again, no evidence of interaction was observed (p > 0.05) (data not shown).

## Discussion

This study found that low socioeconomic position during childhood, youth, and adulthood was associated with higher all-cause mortality among ELSA-Brasil participants over approximately 15 years of follow-up, even after adjusting for sociodemographic factors. Similarly, unfavorable socioeconomic trajectories − whether by risk accumulation or intergenerational social mobility − were linked to an increased risk of death. Notably, upward socio-occupational mobility remained associated with a comparatively smaller increase in mortality risk relative to the high-stable group, even after adjusting for race/skin color. 

Our results confirm that low socioeconomic position during childhood, youth, and adulthood tends to increase all-cause mortality in different populations ^26^ ^,^ ^27^ ^,^ ^28^. In a large cohort based in the United States, exposure to poverty in childhood nearly doubled the risk of premature death ^29^. Robust evidence shows that low socieconomic position in childhood and youth adversely affects educational and occupational trajectories, influencing adult socioeconomic position, promoting unhealthy behaviors, and increasing the risk of conditions such as cardiovascular diseases, type 2 diabetes, and cancer in adulthood ^30^ ^,^ ^31^. Epigenetic pathways also link socioeconomic conditions in childhood and youth to immune system dysregulation and inflammation-related diseases ^30^ ^,^ ^32^.

The association between adult socioeconomic conditions and all-cause mortality is the strongest and most consistently documented in the literature ^33^ ^,^ ^34^. Adulthood is the life stage when individuals complete their education − a key determinant of future exposure to occupational and environmental risks and health-related behaviors ^26^. The *Whitehall Study*, in 24 years of follow-up, found a strong association between low occupational status and a higher incidence of multimorbidity, frailty, and disability in people aged 50 and over − conditions known to be associated with a higher risk of death ^35^. A recent study of six European cohorts identified a higher risk of death among individuals living in poorer areas, with even greater risk observed among those with lower educational levels ^36^.

Our study also found a dose-response relationship between the burden of exposure to low socioeconomic position across the life course and higher risk of death, evidencing that socioeconomic position estimated at a single time point is insufficient to capture its deleterious effects on mortality. In a Scottish cohort of employed women, a composite measure of lifetime socioeconomic experience proved to be a stronger predictor of both all-cause mortality and cardiovascular mortality than other socioeconomic position measures, even after adjusting for age and important proximal intermediate risk factors ^37^. A community-based cohort of older Australians also reported increased risk of death among individuals with cumulative and persistent exposure to disadvantaged socioeconomic conditions throughout life ^8^. Moreover, individuals in the lowest cumulative socioeconomic status group were more than twice as likely to die from cardiovascular diseases than those in the highest life-course socioeconomic position group, according to the *English Longitudinal Study of Ageing* (ELSA) ^38^. Among other reasons, stress generated by chronic exposure to low socioeconomic position leads to increased inflammatory responses, impaired immune function, and accelerated aging. These effects manifest progressively in cells, tissues, and organs, increasing susceptibility to illness and premature mortality ^34^ ^,^ ^39^. A mediation analysis from a South Korean study demonstrated a direct effect of socioeconomic position on mortality and indirect pathways through allostatic load − a cumulative measure of physiological dysregulation − and health behaviors ^40^. 

We tested and found that adverse intergenerational mobility in education and occupation, represented by the downward and low-stable categories, increased the risk of death compared to individuals who maintained a high socioeconomic position. A study in Sweden similarly found a higher risk of death, especially from potentially preventable causes, among individuals who either remained in a low socioeconomic position or experienced downward mobility. In contrast, upward social mobility was not statistically associated with increased risk of death relative to those in stable-high socioeconomic position. These results remained robust even after adjusting for family and genetic factors, including analyses with monozygotic and dizygotic controls ^41^. 

In our study, even individuals who experienced upward occupational mobility in relation to their parents had a higher risk of death compared to those in a high-stable occupational social class, although the magnitude of this association was lower than that observed among those who experienced downward mobility or remained in a low socioeconomic position. These results differ from the *Framingham Heart Study*, which showed decreased mortality and slower aging associated with upward educational mobility ^42^. Similarly, in the *Uppsala Birth Cohort Study*, intragenerational upward mobility appeared to offer protection against mortality from a wide range of causes ^27^. In the *UK Household Longitudinal Study*, upward mobility was associated with a slower pace of aging, although in comparison to individuals who remained disadvantaged throughout life. Thus, our results do not contradict those of the British study, since the HR associated with upward mobility in our analysis was much lower than that observed for downward or high-stable mobility ^9^.

Upward mobility reflects opportunities for social development, whose benefits may help mitigate the effects of adverse early-life exposures ^43^. However, upward mobility does not guarantee equivalent outcomes to those of individuals who have always been in the highest social class ^44^. A study on the consequences of intergenerational upward social mobility suggests that such gains often occur at the expense of adapting to stressful environments, greater workloads, discrimination by the destination social class, among other factors that are detrimental to health ^45^. Therefore, individuals who ascend reach an intermediate health status: better than that of their class of origin but still below that of their destination class. This finding agrees with evidence from DNA methylation studies indicating that early exposure to low socioeconomic position represents a sensitive period, leaving persistent biological and social imprints ^9^. In the *UK 1946 National Survey of Health and Development* birth cohort study, disadvantaged childhood social class, independently of adult socioeconomic position, was associated with accelerated multimorbidity trajectories from age 53 years onwards ^46^.

Race/skin color emerged as an important confounding factor in the association between socioeconomic position and all-cause mortality in our study, as HR substantially reduced after adjustment. Although our findings reinforce the contribution of socioeconomic position to racial health inequities, we found no evidence of interaction between race/skin color and socioeconomic position regarding risk of death. In Brazil, black and mixed-race individuals − who constitute the majority of those living in poverty and with fewer opportunities for upward social mobility ^10^ ^,^ ^47^ − experience worse health outcomes ^10^ ^,^ ^48^ and a higher risk of death from various causes ^49^. During the COVID-19 pandemic, being black or mixed-race was the second greatest risk factor for mortality, surpassed only by age ^50^. The root of these disparities lies in structural racism, which systematically allocates resources and opportunities in favor of white individuals with higher incomes, thereby limiting the chances for social mobility and improvements in health outcomes for black people and low-income populations ^51^. In this system, racism feeds policies and society norms while being strengthened by State structures, perpetuating racial and social disparities in health ^52^.

As illustrated thus far, socioeconomic position is associated with several diseases through multiple mechanisms, which is one of the main reasons why it is a fundamental cause of diseases and health inequalities. Socioeconomic position determines access to various resources (financial, material, social, prestige, etc.), which can be used in different ways to prevent risks or to cope with the disease once it occurs ^15^ ^,^ ^53^. For example, even when a cancer screening test is universally available, demand and access are higher among people with higher levels of education ^53^. Another recent example, during the COVID-19 pandemic, demonstrated that people with better financial conditions were more likely to comply with preventive measures, such as social isolation and the use of personal protective equipment, and had lower mortality rates ^54^. Regardless of changes in risk factors and disease characteristics, socioeconomic position will continue to influence population health outcomes, as it underlies the unequal distribution of resources.

The strengths of this study include its large sample size and a long follow-up period in a middle-income country known for offering limited opportunities for social mobility and a scarcity of evidence on the relationship between life course socioeconomic position, social mobility, and mortality. By examining three life stages (childhood, youth, and adulthood), we were able to capture particularities of each stage on all-cause mortality. We also tested risk accumulation models and intergenerational mobility in education and occupation, approaches that remain underexplored in the literature on socioeconomic position and mortality. Finally, we used educational level and occupational class as socioeconomic position indicators, which are widely disseminated and consolidated in life course epidemiology. 

Some limitations should be considered when interpreting our results. The ELSA-Brasil cohort consists of civil servants from federal education and research institutions, excluding individuals at the extremes of the social hierarchy. This limits the variability of socioeconomic position observed in the sample compared to the general Brazilian population and may underestimate the full association between socioeconomic position and mortality. However, representativeness is not necessary to draw valid inferences about potentially causal associations in well-designed epidemiological studies ^55^. Furthermore, the question on maternal education did not specify a reference period, making it possible that some mothers increased their educational level after the participant’s infancy. This could result in an overestimation of childhood socioeconomic position, potentially attenuating the observed associations. Finally, to compose the cumulative score, we assumed that exposure to low socioeconomic position at different life stages has equal effect on mortality. This may not accurately reflect the differential impact of adversities across the life course.

## Conclusion

Our study showed that exposure to low socioeconomic position at different life course stages, the accumulation of adverse social experiences, and intergenerational downward or stable-low socioeconomic position − and, to a lesser extent, upward mobility − are associated with increased risk of death. Although black and mixed-race individuals tend to experience greater socioeconomic disadvantages throughout life, no evidence was found of interaction between race/skin color and socioeconomic position indicators studied regarding risk of death. These findings corroborate previous studies and reveal the importance of structural social issues in all-cause mortality. Understanding socioeconomic position as a fundamental cause of health inequalities highlights the urgency of addressing socioeconomic inequalities at all life stages. While our findings support the three life course epidemiology models, they strongly indicate that the accumulation of disadvantaged socioeconomic position throughout life has the most powerful impact on mortality risk.
