# Supplementary material for: Life course socioeconomic position, intergenerational social mobility, and mortality among Brazilian public servants in the ELSA-Brasil cohort
Source: Cad Saude Publica. 2025 Oct 24;41(9):e00009625. doi: 10.1590/0102-311XEN009625 (PMC12600023; doi:10.1590/0102-311XEN009625)
Supplement: Supplementary Material [file 1678-4464-csp-41-09-EN009625-s.pdf]

Supplementary material

Figure S1 Intergenerational social mobility matrix (%).

Household head's occupational social class	Current occupational social class							
	Low-low	Low-high	Middle-low	Middle-middle	Middle-upper	High-low	High-upper	Total
Low-low	5.92	2.28	4.11	2.80	0.60	3.24	0.12	19.07
Low-high	5.63	3.42	7.03	6.78	1.52	6.09	0.22	30.69
Middle-low	1.55	1.12	2.32	2.64	0.56	2.78	0.11	11.08
Middle-middle	0.73	0.51	1.30	1.79	0.32	2.81	0.10	7.56
Middle-upper	0.49	0.70	1.34	1.85	0.53	4.71	0.10	9.72
High-low	0.18	0.37	0.62	1.17	0.40	4.80	0.12	7.66
High-upper	0.45	0.70	1.44	2.12	0.83	8.52	0.19	14.25
Total	14.95	9.1	18.16	19.15	4.76	32.95	0.96	100.00

Note: High-stable (green cells): diagonal, middle-middle, middle-upper or high socioeconomic position on both occasions; Upward (blue cells): above diagonal; Downward (yellow cells): below diagonal; Low-stable (red cells): middle-low or low socioeconomic position on both occasions. % referred to N = 13,652.
